# Supplementary material for: WordCommentsAnalyzer: A windows software tool for qualitative research
Source: F1000Res. 2018 Sep 4;7:536. Originally published 2018 May 3. [Version 2] doi: 10.12688/f1000research.14819.2 (PMC6137411; doi:10.12688/f1000research.14819.2)
Supplement: Supplementary file 3 [file f1000research-7-17614-s0002.tgz › b2a79496-4ada-47c4-9682-a99ce867836d.zip!/Eight Iranian Health Journals/Journal of Research in Rehabilitation Sciences _30.docx]

© Journal of Research in Rehabilitation Sciences . This work is licensed under CC BY-NC 4.0 (https://creativecommons.org/licenses/by-nc/4.0/).

Volume & Issue: دوره 9، شماره 3: 1392

Title: ارتباط درد و ترس از درد با ثبات پوسچرال در بیماران مبتلا به کمردرد مزمن غیر اختصاصی

http://jrrs.mui.ac.ir/index.php/jrrs/article/view/875

مقدمه: ترس از درد با ناتوانی و مزمن شدن کمردرد مرتبط است. هدف این مطالعه، بررسی ارتباط ترس از درد با پارامترهای ثبات پوسچر و تأثیر آموزش اختصاصی بر ترس از درد و ثبات پوسچرال در بیماران مبتلا به کمردرد مزمن غیر اختصاصی بود. مواد و روش‌ها: در مطالعه نیمه تجربی حاضر، 27 نفر از زنان مبتلا به کمردرد مزمن غیر اختصاصی بررسی شدند. شدت درد، ترس از درد و ناتوانی با پرسش‌نامه و پارامترهای کنترل پوسچر با صفحه نیرو اندازه‌گیری گردید. همچنین برای اندازه‌گیری تحمل عضلات شکم و کمر به ترتیب آزمون‌های Sit Up و Surensen مورد استفاده قرار گرفت. کلیه آزمون‌ها به فاصله یک هفته و یک ماه پس از یک جلسه آموزشی کوتاه در مورد ترس از درد تکرار شدند. براي مقایسه متغیرها از آزمون Repeated measures و برای بررسی ارتباط متغیرها از آزمون همبستگی Pearson و Mixed Models استفاده شد. یافته‌ها: پس از مداخله شدت درد، ترس از درد و ناتوانی به طور معنی‌داری کاهش یافت (01/0 > P) و مدت زمان ایستادن روی یک پا و تحمل عضلات شکم و کمر افزایش معنی‌داری داشت (01/0 > P). میزان جابه‌جایی مرکز ثقل و سرعت این جابه‌جایی پس از مداخله کاهش معنی‌داری را نشان داد (01/0 > P). شدت درد با جابه‌جایی و سرعت جابه‌جایی مرکز ثقل در حالت ایستادن روی یک پا نیز ارتباط معنی‌داری داشت (01/0 > P)، اما ترس از درد با هیچ یک از پارامترهای ثبات پوسچرال ارتباط معنی‌داری نداشت؛ در حالی که تغییرات ترس از درد با تغییرات پارامترهای جابه‌جایی و سرعت جابه‌جایی مرکز ثقل در صفحه داخلی- خارجی در حالت ایستادن روی یک پا ارتباط معنی‌داری داشت (04/0 = P). نتیجه‌گیری: تغییرات ترس از درد با تغییرات پارامترهای ثبات پوسچرال در حالت ایستادن روی یک پا ارتباط معنی‌داری داشت. کاهش ترس از درد از طریق آموزش اختصاصی در این رابطه موجب کاهش شدت درد، ترس از درد و ناتوانی شده و همچنین موجب بهبود ثبات پوسچرال، افزایش تحمل عضلات کمر و شکم و افزایش زمان حفظ تعادل هنگام ایستادن روی یک پا می‌شود.

Title: بررسی و مقایسه شاخص معلولیت ناشی از اختلال صوتی در معلمان و کاربران غیر حرفه‌ای مبتلا به اختلال صوتي

http://jrrs.mui.ac.ir/index.php/jrrs/article/view/1038

مقدمه: معلمان گروهی از کاربران حرفه‌ای صوت هستند که نسبت به غیر معلمان مشکلات صوتی بیشتری را تجربه می‌کنند و این مشکلات روی سایر جنبه‌های زندگی آن‌ها اثر گذاشته و موجب غیبت از محل کار و ناتوانی شغلی آنان می‌شود. اختلال صوتی به عنوان ترکیبی از علایم خودگزارش شده بیمار و نشانه‌های مشاهده شده بالینی می‌باشد. میزان معلولیتی که بر اثر اختلال صوتی توسط بیمار گزارش می‌شود به شغل، محیط کار، محیط زندگی و واکنش دوستان و اعضای خانواده بستگی دارد. مطالعاتی که معلولیت ناشی از اختلال صوتی را در مشاغل مختلف بررسي می‌کنند، نشان دهنده یافته‌های متضاد و متنوعي می‌باشد. در این مطالعه شاخص معلولیت صوتی در معلمان و کاربران غیر حرفه‌ای صوت مبتلا به اختلال صوتی بررسی و مقایسه شد. مواد و روش‌ها: مطالعه حاضر از نوع توصیفي- تحلیلی- مقطعی بود که بر روي 64 نفر، 32 معلم مبتلا به اختلال صوتی و 32 کاربر غیر معلم مبتلا به اختلال صوتی متوسط تا شديد صورت گرفت. افرادی که به مشکل صوتی خود آگاهی داشتند وارد مطالعه شدند و از ارزیابی آکوستیک برای اطمینان از وجود اختلال صوتي استفاده گردید و آن‌گاه هر دو گروه پرسش‌نامه مربوط به معلولیت ناشی از اختلال صدا (Voice handicap index یا VHI) را تکمیل کردند. داده‌ها با استفاده از روش‌هاي آماری توصیفی و تحلیلی (میانگین و آزمون Independent t و ضریب همبستگی Pearson) مورد تحليل قرار گرفت. یافته‌ها: میانگین نمره کلی VHI در معلمان مبتلا به اختلال صوتی (2/32) نسبت به کاربران غیر حرفه‌ای صوت (3/23) به طور معنی‌داری بیشتر بود (01/0 = P). بین سابقه تدریس و نمره VHI همبستگی وجود داشت. در گروه معلمان و کاربران غیر حرفه‌ای بین دو جنس مرد و زن تفاوتی در نمرات VHI وجود نداشت. نتیجه‌گیری: اختلال صوتي با علایم باليني به نسبت یکسان در مشاغل مختلف تأثیرات متفاوتی ایجاد می‌کند. در معلمان که گروه بسیار بزرگی از کاربران حرفه‌ای صدا هستند، عواقب اختلال صوتی محدودیت بیشتری در زندگیشان ایجاد می‌کند. بنابراین توجه به این امر در برنامه‌ریزی درمانی ضرورت ویژه‌ای دارد.

Title: بررسی تأثیر تقارن در تحمل وزن با استفاده از کفي بر پاسخ‌های پوسچرال عضلات اندام‌های تحتانی در بیماران مبتلا به همی‌پارزی

http://jrrs.mui.ac.ir/index.php/jrrs/article/view/1074

مقدمه: یکی از مشخصات بارز اختلال در کنترل پوسچر در بیماران همی‌پارزی متعاقب سکته مغزی، عدم تقارن در تحمل وزن بر اندام‌های تحتانی می‌باشد. نظر به اهمیت تأثیر فشار وارد شده بر اندام‌ها بر تصحیح رفلکس‌های پوسچرال، در این مطالعه تأثیر کفی بر تقارن تحمل وزن و نیز تأثیر افزایش بار بر اندام مبتلا بر پاسخ پوسچرال عضلات اندام‌های تحتانی در برابر یک اغتشاش داخلی مورد بررسی قرار گرفت. مواد و روش‌ها: 27 بیمار همی‌پارزی متعاقب سکته مغزی که همگی قادر بودند حداقل یک دقیقه بدون استفاده از وسایل کمکی بایستند در این بررسی شرکت نمودند. ارزیابی‌ها شامل اندازه‌گیری شاخص تقارن و نیز زمان تأخیر و شدت پاسخ عضلات پوسچرال اندام‌های تحتانی بود که قبل و پس از قرار دادن یک کفی به ضخامت 10 میلی‌متر زیر اندام تحتانی سالم بیماران ثبت و مورد مقایسه قرار ‌گرفت. جهت اندازه‌گیری شاخص تقارن از بیمار خواسته شد به مدت 20 ثانیه روی صفحه نیرو بایستد. جهت بررسی پاسخ‌های پوسچرال از ثبت الکترومایوگرافی برای عضلات گاسترکنموس داخلی، سولئوس و همسترینگ خارجی اندام‌های تحتانی سالم و مبتلا استفاده شد. از حرکت فلکسیون اندام فوقانی سالم به عنوان یک اغتشاش درونی و به منظور تحریک پاسخ عضلات پوسچرال استفاده شد. یافته‌ها: تأثیر کفی بر بهبود شاخص تقارن معنی‌دار بود (001/0 = P)، اما افزایش بار بر اندام مبتلا، تأثیری بر زمان تأخیر و شدت پاسخ‌های پوسچرال عضلات اندام‌های تحتانی سالم و مبتلا نداشت. نتیجه‌گیری: قرار دادن کفی با نزدیک کردن مرکز گرانش به خط وسط بدن باعث بهبود تقارن تحمل وزن می‌شود. نتایج تحقیق حاضر حاکی از آن است که کاهش بار بر اندام تحتانی مبتلا متعاقب سکته مغزی نمی‌تواند به طور کامل توجیه کننده وجود پاسخ‌های پوسچرال با شدت کم و زمان تأخیر بالا باشد.

Title: تاثیر تمرین دو تکنیک دست در کاراته برکاهش حرکات کلیشه‌ای دست یک پسر اتیستیک خاص: پژوهش موردی

http://jrrs.mui.ac.ir/index.php/jrrs/article/view/814

مقدمه: رفتارهای کلیشه‌ای یکی از ویژگی‌های مهم کودکان مبتلا به اختلال اوتیسم (Autism) می‌باشد. این رفتارها با نقصان در فرایند یادگیری، اکتساب مهارت‌های اجتماعی، افرایش استرس در خانواده و رفتارهای خودآزار دهنده مرتبط می‌باشند. بنابراین درمان این رفتارها از اهمیت بسزایی برخوردار است. هدف اصلی از پژوهش حاضر، تأثیر آموزش دو تکنیک دست در کاراته به بهبود حرکات کلیشه‌ای دست یک پسر اوتیستیک بود. مواد و روش‌ها: شرکت کننده پژوهش حاضر یک پسر 10 ساله مبتلا به اختلال اوتیسم بود. مرحله مداخله که در آن شرکت کننده به تمرین دو تکنیک رزمی دست (3 ماه، 36 جلسه) به همراه 14 کودک در روزهای فرد می‌پرداخت، قبل و بعد از دو مرحله خط پایه که بدون تمرین صورت می‌گرفت، اعمال گردید. فراوانی بروز حرکات کلیشه‌ای دست توسط مشاهده کننده در دوره‌های 15 دقیقه‌ای بلافاصله قبل و بعد از تمرین در حالی که شرکت کننده مشغول به فعالیت‌های منظم کلاسی بود، مورد بررسی قرار گرفت. مشاهده همچنین در روزهای بدون تمرین در دوره‌های زمانی مشابه در تمامی مراحل انجام گرفت. یافته‌ها: آموزش دو تکنیک دست کاراته به کاهش 12/44 درصدی حرکات کلیشه‌ای دست کودک اوتیستیک منجر شد. نتیجه‌گیری: تمرین دو تکنیک دست در کاراته می‌تواند منجر به کاهش چشمگیر حرکات کلیشه‌ای دست کودکان مبتلا به اختلال اوتیسم شود. بنابراین بر پایه یافته این پژوهش پیشنهاد می‌گردد شرکت در رشته‌های رزمی در برنامه‌ هفتگی کودکان مبتلا به اختلال ا‌وتیسم که دارای حرکت کلیشه‌ای دست هستند، قرار گیرد.

Title: بررسی و مقایسه فراوانی ناروانی در کلمات و ناکلمات در کودکان مبتلا به لکنت

http://jrrs.mui.ac.ir/index.php/jrrs/article/view/892

مقدمه: لکنت اختلالی چند بعدی می‌باشد و عوامل بسیاری بر آن تأثیرگذار است. یکی از عوامل مؤثر بر لکنت جنبه‌های زبانی می‌باشد. هدف اين پژوهش، بررسي فراواني بروز نارواني در كلمات و ناكلمات در کودکان مبتلا به لكنت بود. مواد و روش‌ها: مطالعه حاضر از نوع توصیفی- تحلیلی و مقایسه‌ای بود. 15 کودک 5-4 ساله مبتلا لکنت در این مطالعه شرکت داشتند. از کودکان خواسته شد تا کلمات و ناکلماتی را که می‌شنوند، تکرار نمایند. نمونه‌ها جهت جمع‌آوری و تحلیل داده‌ها ضبط گردید و مورد برررسی قرار گرفت. تحلیل آماری داده‌ها توسط نرم‌افزار SPSS انجام شد. یافته‌ها: فراوانی ناروانی در کلمات و نیز ناکلمات به طور معنی‌داری متفاوت بود و بروز ناروانی حین تکرار در ناکلمات بیشتر از کلمات مشاهده شد (05/0 > P)، همچنین تكرار و قفل نارواني‌ها شايع بودند. نتیجه‎گیری: به طور کلی نتایج پژوهش حاضر نشان داد که پیچیدگی‌های زبان‌شناختی باعث افزایش بسامد وقوع ناروانی می‌شود.

Title: وضعیت قرارگیری پا در بازیکنان بسکتبال دارای سابقه ابتلا به شین اسپلینت

http://jrrs.mui.ac.ir/index.php/jrrs/article/view/1033

مقدمه: شین اسپلینت یکی از آسیب‌های شایع در ورزشکاران می‌باشد. مطالعات مختلف ارتباط بین وضعیت قرارگیری (پوسچر) پا و این آسیب را نشان داده‌اند. هدف از این مطالعه، ارزیابی قرارگیری پا در ورزشکاران رشته بسکتبال دارای سابقه ابتلا به شین اسپلینت بود. مواد و روش‌ها: در مجموع 30 ورزشکار رشته بسکتبال که در طی 3 ماه گذشته سابقه ابتلا به شین اسپلینت را داشتند، در این تحقیق شرکت کردند. به منظور ارزیابی وضعیت قرارگیری پای ورزشکاران از مقیاس معتبر پوسچر پا (Foot posture index) که دارای شش بخش می‌باشد، استفاده شد. وضعیت قرارگیری سر استخوان قاپ، انحناهای فوقانی و تحتانی قوزک خارجی، اينورشن يا اورشن پاشنه، برجستگی اطراف مفصل قاپ- ناوي، راستاي قوس طولي داخلي پا و نزديک شدن يا دور شدن پنجه پا نسبت به پشت پا، شش بخشی بودند که به وسیله این مقیاس در حالت ایستاده مورد اندازه‌گیری قرار گرفتند و پوسچر پای فرد به عنوان طبیعی، سوپينيشن و پرونيشن طبقه‌بندي شد. یافته‌ها: در مجموع یافته‌های پوسچر پای 30 بسکتبالیست شرکت کننده در این تحقیق نشان داد که اکثریت شرکت کنندگان (80 درصد) دارای پوسچر پای غیر طبیعی بودند. 63 درصد شرکت کنندگان دارای پرونیشن پا (53 درصد پرونیشن و 10 درصد پرونیشن بیش از حد پا) بودند. پوسچر پای 17 درصد شرکت کنندگان در وضعیت سوپینیشن بود. همچنین نتایج این مطالعه تفاوت معنی‌داری را بین پوسچر پای راست و چپ آزمودنی‌ها نشان نداد (05/0 < P). نتیجه‌گیری: به طور کلی میزان ناهنجاری‌های پوسچر پا در ورزشکاران رشته بسکتبال دارای سابقه ابتلا به شین اسپلینت بالا بود. با توجه به این‌که مطالعات دیگر ارتباط معنی‌داری بین بروز شین اسپلینت و ناهنجاری‌های پا را گزارش‌کرده‌اند، می‌توان نتیجه گرفت که ممکن است یکی از علل بروز این آسیب در بسکتبالیست‌ها، وضعیت پوسچر پای غیر طبیعی آن‌ها باشد. از این رو مربیان باید در خصوص اصلاح این ناهنجاری‌ها تمهیداتی مانند استفاده از کفش‌ها و کفی‌های مخصوص بیاندیشند.

Title: مقايسه تأثير تمرینات بدنی، ذهنی و ذهنی- بدنی بر قابليت تعادل دانش‌آموزان نابينا

http://jrrs.mui.ac.ir/index.php/jrrs/article/view/782

مقدمه: هدف پژوهش حاضر، مقايسه تأثير تمرینات بدنی، ذهنی و ذهنی- بدنی بر قابليت تعادل دانش‌آموزان نابينا بود. مواد و روش‌ها: شرکت کنندگان پژوهش تجربی حاضر، 30 دختر (با میانگین سن گروه تمرينات ذهني 2/9 سال، گروه تمرينات بدني 2/8 سال و گروه تمرينات ذهني- بدني 6/9 سال) با تیزی دید حداکثر 05/0 دیوپتر در چشم برتر (با استفاده از عینک) بودند که به روش نمونه‌گیری تصادفي ساده به سه گروه ده نفره (تمرینات بدنی، تمرین ذهنی و تمرین ترکیبی (ذهنی- بدنی) تقسیم شدند. مدت زمان مداخله گروه‌ها 8 هفته دو جلسه‌ای در رژیم زمانی 45 تا 60 دقیقه در هر جلسه بود. قابلیت تعادل ایستا و پویا به ترتیب توسط آزمون‌های Romberg و تخته تعادل اندازه‌گیری شد. تحلیل آماری داده‌ها با استفاده از نرم‌افزار SPSS نسخه 18 و از طریق آزمون‌های t Independent، ANOVA و آزمون تعقیبی Tokey صورت گرفت (سطح معنی‌داری 95 درصد و میزان 05/0 £ a). یافته‌ها: در تعادل ایستا تفاوت معنی‌داری میان گروه‌ها وجود نداشت، ولي در تعادل پويا، تنها روش تمرین ترکیبی موجب توسعه قابلیت تعادل پويا شد. به علاوه، مقایسه‌های بین گروهی برتری معنی‌دار این روش نسبت به هر یک از روش‌های تمرین ذهنی و بدنی را نشان داد. نتیجه‌گیری: عدم وجود تفاوت معنی‌دار در متغیر تعادل ایستا میان گروه‌های سه‌گانه نشان دهنده ساز و کارهای اصلاحی و جبرانی است، ولي در فعالیت‌هايی كه با حركت توأم است و پیچیده‌تر مي‌گردد، برای رشد قابلیت تعادل بهتر است ترکیب تمرینات ذهنی و بدنی به کار رود.

Title: مقايسه استقامت عضلات ثبات دهنده کمری- لگنی در زنان ورزشكار مبتلا و غیر مبتلا به سندرم درد پاتلافمورال

http://jrrs.mui.ac.ir/index.php/jrrs/article/view/766

مقدمه: گزارش شده است که راستای نامناسب اندام تحتانی و درد پاتلافمورال (Patellofemoral pain یا PFP) مرتبط با ضعف عضلات ران می‌باشد. کنترل و ثبات ناحیه مرکزی بدن مستلزم قدرت کافی در عضلات ران و تنه و هماهنگی بین آن‌ها است. هدف از انجام این تحقیق، مقايسه استقامت عضلات ثبات دهنده کمری- لگنی در زنان ورزشكار مبتلا و غیر مبتلا به سندرم درد پاتلافمورال بود. مواد و روش‌ها: در تحقیق حاضر 30 ورزشکار زن مبتلا به سندرم درد پاتلافمورال با میانگین و انحراف استاندارد سنی 75/2 ± 60/21 سال، قد 073/0 ± 165 سانتی‌متر و وزن70/8 ± 70/60 کیلوگرم و 30 ورزشکار زن غیر مبتلا با میانگین و انحراف استاندارد سنی 63/2 ± 77/20 سال، قد 063/0 ± 164 سانتی‌متر و وزن66/6 ± 53/60 کیلوگرم در رشته‌های والیبال، بسکتبال و هندبال در سطح باشگاهی شرکت کردند. اندازه‌گیری استقامت عضلات ثبات دهنده کمری- لگنی در چهار طرف با استفاده از آزمون‌های عملکردی McGill شامل آزمون خم کردن تنه، آزمون باز کردن تنه (آزمون تعدیل شده Biering-Sorensen) و آزمون پل زدن به چپ و راست انجام شد. برای تجزیه و تحلیل داده‌ها از آمار توصیفی Independent t در برنامه SPSS نسخه 13 با سطح معنی‌داری 05/0 = P استفاده گردید. یافته‌ها: بین استقامت عضلات ثبات دهنده کمری- لگنی در گروه مبتلا به سندرم درد پاتلافمورال و گروه غیر مبتلا تفاوت معنی‌داری وجود داشت (001/0 = P) و بیشترین تفاوت بین دو گروه در استقامت عضلات ثبات دهنده قدامی تنه (عضلات شکم) مشاهده شد. با توجه به نتایج تحقیق احتمال می‌رود زنان جوان مبتلا به سندرم درد پاتلوفمورال دارای استقامت کمتری در عضلات ثبات دهنده کمری- لگنی نسبت به زنان سالم باشند. نتیجه‌گیری: با توجه به نتایج تحقیق، تمرینات استقامتی عضلات ثبات دهنده کمری- لگنی جهت پیشگیری و یا به عنوان یکی از اجزای درمان سندرم درد پاتلافمورال در افراد مشابه با شرایط و ویژگی‌های نمونه‌های تحقیق حاضر پیشنهاد می‌شود.

Title: مقایسه توجه پایدار به محرک شنیداری و دیداری در کودکان مبتلا به اختلال یادگیری و همتایان سالم

http://jrrs.mui.ac.ir/index.php/jrrs/article/view/938

مقدمه: کودکان مبتلا به اختلال یادگیری (Learning disorders یا LD) در یک یا چند فرایند اساسی روانی دخیل در فهمیدن یا کاربرد زبان شفاهی یا کتبی اختلال نشان می‌دهند. هدف از مطالعه حاضر، مقایسه توجه پایدار به محرک شنیداری و دیداری در کودکان مبتلا به اختلال یادگیری نامعین و همتایان سالم بود. مواد و روش‌ها: در این مطالعه 26 کودک دبستانی مبتلا به اختلال یادگیری نامعین و 40 کودک دبستانی سالم مورد بررسی قرار گرفتند. جهت جمع‌آوری داده‌ها، آزمون هوش Wechsler کودکان (Wechsler intelligence scale for children یا WISC) و آزمون عملکرد مداوم بینایی و شنیداری به کار برده شد. برای تحلیل آزمون‌ها نیز آزمون Paired t یا Wilcoxon و Independent t یا Mann-Whitney توسط نرم‌افزار SPSS نسخه 16 به کار گرفته شد. یافته‌ها: دو گروه در موضوع توجه پایدار بینایی یا خطای درجاماندگی (003/0 = P)، تفاوت میانگین خطای ارتکاب یا تکانشگری در برابر محرک دیداری و شنیداری (005/0 £ P) و میانگین زمان پاسخ به محرک دیداری و شنیداری (005/0 £ P) تفاوت معنی‌دار داشتند. نتايج همچنين عدم تفاوت معنی‌دار (752/0 = P) را بین توجه پایدار شنیداری یا همان درجاماندگی در دو گروه نشان داد. نتیجه‌گیری: بر اساس نتايج مطالعه حاضر به نظر مي‌رسد کودکان مبتلا به اختلال یادگیری دچار نقص توجه پایدار می‌باشند. همچنین گروه مبتلا در توجه انتخابی شنیداری و کنترل تکانشگری در برابر محرک شنیداری دچار نقصان بودند. در نهایت این‌که کودکان مبتلا به اختلال یادگیری در برابر محرک‌های بینایی نسبت به نوع شنیداری بهتر عمل کردند، بنابراین استفاده از محرک بینایی در جلسات توان‌بخشی و آموزش این کودکان توصیه می‌شود.

Title: اثربخشی توان‌بخشی روانی به شیوه Dohsa-hou بر کاهش میزان شدت خستگی، افسردگی، اضطراب، استرس و ارتقای کیفیت زندگی بیماران مبتلا به مالتیپل اسکلروزیس (MS) شهر اصفهان

http://jrrs.mui.ac.ir/index.php/jrrs/article/view/958

مقدمه: بیماران مبتلا به مالتیپل اسکلروزیس (Multiple sclerosis یا MS) به دلیل علایم جسمی- روانی و عدم قبول واقعیت‌ها دچار علایم خلقی منفی می‌شوند و همراه شدن خستگی مزمن با خلق منفی بر کیفیت زندگی آنان تأثیر منفی می‌گذارد. بنابراین پژوهش حاضر با هدف، بررسی اثربخشی توان‌بخشی روانی به شیوه Dohsa-hou بر کاهش میزان شدت خستگی، افسردگی، اضطراب، استرس و ارتقای کیفیت زندگی در بیماران مبتلا به مالتیپل اسکلروزیس شهر اصفهان انجام شد. مواد و روش‌ها: پژوهش حاضر از نوع نیمه تجربی با طرح پیش‌آزمون- پس‌آزمون و پیگیری با گروه شاهد بود. جامعه آماری تحقیق را کلیه بیماران مبتلا به MS شهر اصفهان تشکیل می‌دادند که با استفاده از روش نمونه‌گیری در دسترس تعداد 30 بیمار در قالب دو گروه آزمایش (15 نفر) و شاهد (15 نفر) انتخاب شدند. بیماران قبل از آغاز دوره درمان به تکمیل پرسش‌نامه‌های مقیاس شدت خستگی (Fatigue severity scale یا FSS)، مقیاس افسردگی- اضطراب و استرس (Depression‚ Anxiety‚ Stress scale-21 یا DASS-21) و پرسش‌نامه کیفیت زندگی بیماران مبتلا به MS (Multiple sclerosis quality of life-54 یا MSQOL-54) مبادرت نمودند و در پایان دوره درمان هم در مرحله پس‌آزمون و هم پیگیری نیز این ارزیابی صورت گرفت. مدت دوره درمان Dohsa-hou در گروه آزمایش 11 جلسه بود که به صورت هر هفته سه جلسه انجام شد و مرحله پیگیری 40 روز بعد از پس‌آزمون صورت گرفت. داده‌های حاصل از پرسش‌نامه با استفاده از نرم‌افزار SPSS نسخه 17 و آزمون‌های توصیفی (میانگین و انحراف استاندارد) و استنباطی (MANOVA) تجزیه و تحلیل گردید. یافته‌ها: یافته‌های حاصل از آزمون MANOVA نشان داد که توان‌بخشی روانی Dohsa-hou باعث کاهش میزان شدت خستگی، افسردگی، اضطراب، استرس و ارتقای کیفیت زندگی بیماران مبتلا به MS شد (001/0 £ P). نتیجه‌گیری: از آن‌جایی که بیماری MS منجر به بروز علایم وسیعی می‌شود و بیماران مبتلا به آن ممکن است در آینده نیاز به توان‌بخشی داشته باشند، روش Dohsa-hou به عنوان روش توان‌بخشی روانی، درمان مؤثری بر کاهش شدت خستگی، افسردگی، اضطراب، استرس و ارتقای کیفیت زندگی این بیماران می‌باشد.

Title: بررسی ارتباط بین امتیازات آزمون‌های غربالگری عملکردی با سابقه آسیب‌ دیدگی و تعیین نمره پیش‌بین غربالگری برای آسیب دیدگی

http://jrrs.mui.ac.ir/index.php/jrrs/article/view/967

مقدمه: اظهار نظر در مورد بازگشت به ورزش متعاقب برخی آسیب دیدگی‌ها در هاله‌ای از ابهام قرار دارد. هدف از اجرای تحقیق حاضر، بررسی ارتباط بین امتیازات آزمون‌های غربالگری عملکردی با سابقه آسیب دیدگی و تعیین نمره پیش‌بین غربالگری برای آسیب دیدگی بود. مواد و روش‌ها: جامعه آماری این تحقیق حدود 100 دانشجو (50 مرد و 50 زن) با میانگین سنی 12/2 ± 21/23 سال بودند. از آزمون‌های غربالگری حرکتی عملکردی (Functional movement screening یا FMS) شامل Deep Squat، گام برداشتن از روی مانع، Lunge، تحرک‌پذیری شانه، بالا آوردن فعال پا، شنای پایداری تنه و پایداری چرخشی برای ارزیابی آزمودنی‌ها استفاده شد. همچنین تجزیه و تحلیل آماری با استفاده از ضریب همبستگی Pearson، Kappa، آزمون t و ANOVA صورت گرفت. در نهایت داده‌ها توسط برنامه SPSS نسخه 17 و در سطح معنی‌داری 05/0 تجزیه و تحلیل شدند. یافته‌ها: بر طبق یافته‌ها، افراد بدون آسیب نمرات FMS بهتری نسبت به افراد آسیب دیده داشتند. تفاوت در نمرات آزمون FMS بین گروه‌های آسیب مچ پا و آسیب زانو با گروه بدون آسیب معنی‌دار بود. نمره Cut-off برای آزمون‌های FMS حدود 17 به دست آمد و استفاده از نسبت احتمال نشان داد که اگر ورزشکاری نمره کمتر از 17 در آزمون‌های FMS کسب کند، حدود 7/4 برابر بیشتر از افراد دارای نمره بالاتر مستعد آسیب‌های اندام تحتانی می‌باشد. نتیجه‌گیری: نمرات آزمون‌های FMS مدل اولیه و دقیق پیشگیری از آسیب را در اختیار مربیان قرار می‌دهد تا این‌که میزان بروز آسیب‌‌های اندام تحتانی را در ورزشکارانشان پیشگویی کنند. بنابراین نیاز است که مربیان تیم‌های دانشگاهی در زمینه پیشگیری از آسیب‌های ورزشی، هم‌راستا با آزمایش‌های پزشکی، آزمون‌های FMS را نیز به عنوان یک ابزار معتبر به کار برند و سطح عملکردی ورزشکارانشان را بسنجند.

Title: بررسی و مقایسه توانایی تکرار ناکلمه در دانش‌آموزان نارساخوان پایه سوم ابتدایی

http://jrrs.mui.ac.ir/index.php/jrrs/article/view/1143

چکیده مقدمه: یکی از شایع‌ترین اختلالات خواندن، نارساخوانی رشدی می‌باشد. بر اساس فرضیه نقص واج‌شناختی، مشکل اصلی این کودکان در پردازش واجی و ایجاد بازنمایی‌های نادرست می‌باشد. تکلیف تکرار ناکلمه رمزگذاری، ذخیره‌سازی و بازیابی بازنمایی‌های واجی را منعکس می‌کند، به همین دلیل تكرار ناكلمه، شاخص مناسبی برای سنجش این مهارت‌ها به خصوص حافظه فعال واجی می‌باشد. همچنین این تکلیف فرایندهای ادراک گفتار، دانش واژگانی، طرح‌ریزی حرکتی و تولید را نیز درگیر می‌کند. بنابراین هدف این مطالعه، بررسی عملکرد دانش‌آموزان نارساخوان در تکلیف تکرار ناکلمه و تحلیل خطاهای آن‌ها بود. مواد و روش‌ها: اين مطالعه غیر تجربی- تحلیلی از نوع مقطعی و مورد- شاهدی بود. دو گروه، 15 دانش‌آموز نارساخوان و 35 دانش‌آموز طبیعی پایه سوم ابتدایی در اين مطالعه شركت كردند. اين دانش‌آموزان نبايد دارای مشکلات عصب شناختی درشت، روان‌پزشکی و اختلالات گفتار و زبان باشند و بايد از هوشبهر و شنوایی طبیعی برخوردار باشند. 40 ناکلمه که شامل ناکلمات يک، دو، سه و چهار هجایی بود، جهت بررسی عملکرد تکرار این گروه استفاده شد. پاسخ‌ها آوانویسی گسترده (Broad) گردید و نتایج با استفاده از آماره‌های توصیفی و تحلیلی مورد تجزیه و تحلیل قرار گرفت. یافته‌ها: گروه نارساخوان نسبت به گروه شاهد، نمره کمتری در تکلیف تکرار ناکلمه کسب کردند و تفاوت بین دو گروه از نظر آماری معنی‌دار بود (001/0 > P). نتیجه‌گیری: خطاهای گروه نارساخوان در تکرار ناکلمه، نشان داد که آن‌ها نقایصی در دسترسی، حفظ و بازیابی کدهای واجی تجربه می‌کنند. همچنین آن‌ها مشکلاتی در تمییز واج‌هایی دارند که به طور ضعیف رمزگذاری شده‌اند.

Title: اثرات تمرین کوتاه‌مدت با ویبره کل بدن بر فعالیت نوروماسکولار عضلات نواحی مهم از لحاظ شکستگی‌های پوکی استخوان در حداکثر فعالیت ایزومتریک ارادی در زنان جوان سالم

http://jrrs.mui.ac.ir/index.php/jrrs/article/view/907

مقدمه: اثر ویبره کل بدن (Whole body vibration یا WBV) بر پاسخ الکترومایوگرافیک عضلات، بیشتر به صورت حاد و فوری و اغلب در نواحی نزدیک به صفحه ویبره مورد مطالعه قرار گرفته و بررسی اثرات بلندمدت‌تر WBV بر پاسخ الکترومایوگرافی عضلات دور از صفحه ویبره، به ویژه با دید درمانی مورد غفلت واقع شده است. هدف از این مطالعه، بررسی پاسخ الکترومایوگرافیک عضلات دور از صفحه ویبره به تحریک ویبره با دید درمانی، در نواحی مهم از نظر شکستگی‌های استخوانی حاصل از بیماری پوکی استخوان و در جمعیت در معرض خطر ابتلا به این بیماری یعنی زنان بود. مواد و روش‌ها: 12 زن جوان سالم غیر ورزشکار (71/2 ± 66/25 سال) در مطالعه طراحی شده به صورت کارآزمایی بالینی یک سوکور شرکت کردند و به طور تصادفی به یکی از گروه‌های ویبره کل بدن (WBV) (تمرین سمی اسکوات استاتیک + ویبره؛ ویبره عمودی، فرکانس 30 هرتز و دامنه قله به قله جابه‌جایی 3 میلی‌متر) و درمان‌نما (تمرین سمی اسکوات استاتیک بدون ویبره) وارد شدند. به استثنای خاموش بودن دستگاه WBV در گروه درمان‌نما، پروتکل تمرین در هر دو گروه یکسان بود. آزمون‌های الکترومایوگرافی عضلات ارکتور اسپاین کمر، گلوتئوس ماگزیموس و رکتوس فموریس قبل و بعد از 12 جلسه تمرین انجام و پاسخ‌های دامنه فعالیت الکترومایوگرافی عضله (Root mean square of Electromyography یا RMS EMG) و فرکانس میانه برای تجزیه و تحلیل استخراج شد. یافته‌ها: پاسخ RMS دو عضله ارکتور اسپاین کمر (017/0 = P)، گلوتئوس ماگزیموس (014/0 = P) و همچنین فرکانس میانه عضله ارکتور اسپاین کمر (020/0 = P) در اثر تمرین WBV نسبت به قبل از تمرین افزایش نشان داد، اما اثر خالص WBV در مقایسه با تمرین درمان‌نما فقط در RMS عضله گلوتئوس ماگزیموس افزایش معنی‌داری داشت (001/0 > P) و این افزایش به طور قابل توجهی بیش از دو عضله دیگر بود (001/0 = P). نتیجه‌گیری: نتایج این مطالعه حاکی از تأثیر مثبت خالص 12 جلسه تمرین با WBV بر دامنه فعالیت الکتریکی عضله گلوتئوس ماگزیموس بود. این عضله دور از صفحه ویبره بود و یکی از عضلاتی است که تقویت آن می‌تواند باعث وارد آوردن نیروهای فیزیولوژیک بر تروکانتر بزرگ فمور و شاید تقویت آن شده و از شکستگی‌های حاصل از بیماری پوکی استخوان در این ناحیه پیشگیری نماید. اهمیت کلینیکی این یافته باید در مطالعات و جمعیت‌های دیگر مانند زنان یائسه بیشتر تحقیق گردد.

Title: روایی و پایایی نسخه فارسی سیاهه اختلال هماهنگی رشد حرکتی در کودکان 3 تا 5 ساله

http://jrrs.mui.ac.ir/index.php/jrrs/article/view/791

مقدمه: مطالعات پیشین به اهمیت شناسایی به هنگام کودکان دارای اختلال هماهنگی رشدی به منظور جلوگیری از بروز مشکلات ثانویه تحصیلی، عاطفی و اجتماعی این اختلال تأکید کرده‌اند. درک بهتر مشکلات مذکور نیاز به یک ابزار اندازه‌گیری معتبر دارد. در این راستا گزارش‌های والدین دارای پتانسیل خوبی به منظور فراهم آوردن اطلاعات تاریخچه‌ای مهارت‌های حرکتی و همچنین درک مشکلات حرکتی کودکانشان می‌باشد. از این رو هدف از انجام این مطالعه، تطبیق و تعیین روایی و پایایی نسخه فارسی سیاهه اختلال هماهنگی رشد حرکتی در ایران برای غربال کودکان در معرض خطر ابتلا به اختلال هماهنگی رشدی بود. مواد و روش‌ها: همسانی درونی، روایی سازه و روایی هم‌زمان این سیاهه با یک نمونه 600 نفری از کودکان 3 تا 5 ساله و والدینشان ارزیابی شد. به منظور بررسی روایی هم‌زمان، نتایج ارزیابی والدین با عملکرد کودکان در آزمون رشد حرکتی درشت-2 مورد مقایسه قرار گرفت. یافته‌ها: پایایی همسانی درونی برای همه سؤالات پرسش‌نامه مذکور، 946/0 به دست آمد. تحلیل عاملی اکتشافی و تأییدی نشان داد که سیاهه اختلال هماهنگی رشدی فارسی چند عاملی است و با نسخه اصلی آن همخوانی دارد؛ به طوری که سه عامل (حرکات ظریف، کنترل در حین حرکت و هماهنگی عمومی) در مجموع 22/74 درصد از کل واريانس را تبيين کردند. همچنین طرح فرض شده به خوبی برای نمونه تحت بررسی برازش شد. در نهایت همبستگی بالایی بین نمرات حاصل از این پرسش‌نامه با دو خرده مقیاس آزمون رشد حرکتی درشت-2، جایجایی (883/0 = r) و کنترل شی‌ء (807/0 = r) به دست آمد. نتیجه‌گیری: بر اساس نتایج، نسخه تهیه شده فارسی برای سیاهه اختلال هماهنگی رشدی را می‌توان به عنوان یک ابزار روا و پایا به منظور غربالگری کودکان در معرض خطر اختلال هماهنگی رشد حرکتی در ایران استفاده کرد.

Title: ارتباط ناهنجاري‌هاي ستون فقرات با دردهاي اسكلتي- عضلاني در دانشجويان دانشگاه علوم پزشکی همدان

http://jrrs.mui.ac.ir/index.php/jrrs/article/view/996

مقدمه: ستون فقرات در فعالیت‌های مختلف حمایت اصلی از بدن را به عهده دارد. ثبات و پوسچر مناسب این ساختار به عملکرد هم‌زمان عضلات و عناصر غیر فعال و همچنین عناصر عصبی بستگی دارد. هدف این مطالعه، بررسی ارتباط بین ناهنجاری‌های ستون فقرات با دردهای اسکلتی- عضلانی در نواحی مرتبط با ناهنجاری در دانشجویان دانشگاه علوم پزشکی همدان بود. مواد و روش‌ها: تعداد 241 دانشجو (157 دختر با میانگین سنی 1/1 ± 42/19 سال و 84 پسر با میانگین سنی 25/2 ± 01/21 سال) به طور داوطلبانه در مطالعه مقطعی حاضر وارد شدند. ستون فقرات با استفاده از صفحه شطرنجی، شاقول و آزمون نیویورک از لحاظ ناهنجاری سرکج، سر به جلو، شانه نامتقارن، کیفوز و لوردوز بررسی شد. محل درد و شدت درد با معیار مشاهده‌ای درد در نواحی سر، ناحیه راست و چپ گردن، ناحیه راست و چپ شانه و کمر با استفاده از یک پرسش‌نامه مورد ارزیابی قرار گرفت. در نهایت داده‌ها با استفاده از ضریب همبستگی مورد تجزیه تحلیل قرار گرفت. یافته‌ها: بين ناهنجاري سركج (در صفحه فرونتال) با سردرد و درد گردن در سمت چپ ارتباط معنی‌داری وجود داشت. (05/0 > P). ارتباط بين كيفوز و درد در ناحيه راست و چپ شانه و درد ناحيه بين دو كتف نيز معنی‌دار بود (05/0 > P). همچنين بين ناهنجاري لوردوز و درد كمر نيز ارتباط معنی‌داري مشاهده شد (05/0 > P). نتیجه‌گیری: نتایج حاصل از این مطالعه رابطه بین وجود برخی از ناهنجاری‌های وضعیتی و بروز دردهای اسکلتی- عضلانی را نشان داد.

Title: تأثیر خستگی موضعی عضلات ارکتوراسپاین بر بازیابی تعادل در افراد مبتلا به کایفوز پس از اعمال آشفتگی پوسچرال در صفحه ساجیتال

http://jrrs.mui.ac.ir/index.php/jrrs/article/view/900

مقدمه: کنترل پوسچر تحت عنوان حفظ اندام‌ها و قسمت‌های مختلف بدن در وضعیت ایستاتیک و دینامیک در راستای بیومکانیکی مناسب تعریف می‌شود. خستگی یکی از عواملی است که وضعیت بدن را تحت تأثیر قرار می‌دهد. خستگی عضلانی می‌تواند اطلاعات رسیده از منابع حسی به مغز را مختل کرده و تعادل را از بین ببرد. بنابراین خستگی می‌تواند بر بازیابی تعادل اثرگذار باشد و فرد را از وضعیت طبیعی خارج کند. بنابراین هدف از مطالعه حاضر، بررسی اثر خستگی موضعی عضلات ارکتوراسپاین بر بازیابی تعادل در افراد مبتلا به کایفوزیس پس از اعمال آشفتگی پوسچرال بود. مواد و روش‌ها: 12 نفر از دانشجویان مرد با انحنای ستون فقرات پشتی بیش از40 درجه (سن: 42/1 ± 25/23 سال، قد: 89/6 ± 92/174 سانتی‌متر و وزن: 17/12 ± 33/67 کیلوگرم) و 12 نفر با انحنای طبیعی ستون فقرات پشتی (سن: 53/1 ± 17/23سال، قد: 16/6 ± 92/178 سانتی‌متر و وزن: 34/9 ± 75/71 کیلوگرم) به شکل همتاسازی در مطالعه شرکت کردند. برای اندازه‌گیری میزان درجه کایفوز پشتی از خط‌کش منعطف استفاده شد. برای ارزیابی تعادل، میزان تغییرات جابجایی و مسافت مرکز فشار پا (Center of pressure یا COP)، با استفاده از اعمال اغتشاش ناگهانی در جهت قدامی- خلفی از دستگاه Foot scan استفاده شد. آزمایش‌ها در دو شرایط قبل و پس از خستگی با رها کردن وزنه‌ای برابر با 10 درصد وزن هر آزمودنی انجام گرفت. تجزیه و تحلیل اطلاعات توسط آزمون‌های ANOVA با اندازه‌گیری‌های مکرر (ANOVA with repeated measures) و Independent t صورت گرفت. یافته‌ها: مسافت جابجایی COP پس از اعمال خستگی در گروه شاهد (685/0 = P) و گروه کایفوتیک (678/0 = P) از لحاظ آماری اختلاف معنی‌داری را نشان نداد. بنابراین خستگی موضعی عضلات ارکتوراسپاین تأثیری بر بازیابی تعادل نداشت. نتیجه‌گیری: به احتمال زیاد بازیابی پوسچر پس از اختلال در تعادل در افراد کایفوتیک تحت تأثیر خستگی عضلات ارکتوراسپاین قرار نمی‌گیرد.

Title: اثر شش هفته تمرینات هاپینگ بر روی حس وضعیت مفصل ورزشکاران دارای بی‌‌ثباتی عملکردی مچ پا

http://jrrs.mui.ac.ir/index.php/jrrs/article/view/965

مقدمه: هدف از پژوهش حاضر، بررسی تأثیر شش هفته تمرینات هاپینگ بر روی حس وضعیت مفصل ورزشکاران جوان دارای بی‌‌ثباتی عملکردی مچ پا بود. مواد و روش‌ها: 30 جوان پسر ورزشکار دارای بی‌ثباتی عملکردی مچ پا با میانگین و انحراف استاندارد وزن 50/11 ± 96/73 کيلوگرم، قد 24/8 ± 95/180 سانتي‌متر، سن 46/1 ± 16/22 سال و نمره بی‌ثباتی 31/2 ± 20 به صورت تصادفی به دو گروه 15 نفری شامل گروه تجربی و گروه شاهد تقسیم شدند. قبل و بعد از اجرای شش هفته برنامه تمرینی، حس وضعیت مفصل مچ پای آزمودنی‌ها از طریق بازسازی زاویه مفصل به صورت فعال اندازه‌گیری گردید. برای تجزیه و تحلیل داده‌ها از روش آمار توصیفی، Independent t و Paired t در سطح معنی‌داری 95 درصد (05/0 ≥ α) استفاده شد. یافته‌ها: تمرینات هاپینگ موجب کاهش معنی‌دار میانگین خطای مطلق بازسازی زاویه مفصل مچ پا در حرکت اینورشن و پلانتار فلکشن بعد از شش هفته تمرینات هاپینگ شد. همچنین کاهش بیشتری در خطای مطلق بازسازی زاویه اینورشن نسبت به پلانتار فلکشن مشاهده شد. نتیجه‌گیری: با توجه به نتایج تحقیق توصیه می‌شود به دلیل نیاز ویژه افراد دارای بی‌ثباتی عملکردی مچ پا به حس وضعیت مفصل در مهارت‌های ورزشی و همچنین به عنوان یک عامل مهم توان‌بخشی در طراحی برنامه‌های تمرینی و بازتوانی از مزایای برنامه تمرین هاپینگ استفاده شود.

Title: بررسی تأثیر سن کاشت حلزون بر مهارت‌هاي خواندن کودکان کم‌شنوا

http://jrrs.mui.ac.ir/index.php/jrrs/article/view/841

مقدمه: کاشت حلزون تأثیرات معنی‌داری بر مهارت‌های زبان، گفتار و خواندن در کودکان ناشنوا دارد. هدف این مطالعه، بررسی تأثیر سن کاشت حلزون در روند پیشرفت مهارت‌هاي خواندن کودکان کاشت شده بود. مواد و روش‌ها: پژوهش حاضر بر روی 24 کودک کاشت حلزون در دو پایه دوم و سوم مقطع ابتدایی انجام شد. دانش‌آموزان از بین مدارس ابتدایی شهر تهران انتخاب شدند و عملکرد خواندن کودکان توسط آزمون خواندن نما مورد ارزیابی قرار گرفت. یافته‌ها: بین مهارت‌های خواندنبا سن کودک در زمان جراحی کاشت و همچنین مدت زمان کاشت حلزون رابطه معنی‌داری دیده شد (05/0 > P) نتیجه‌گیری: کاشت حلزون باعث افزایش درون‌داد شنیداری و در نتیجه بهبود مهارت‌های زبانی و خواندن می‌شود؛ بنابراین لازم است اقدامات لازم جهت جراحی زودهنگام کودکان کم‌شنوا و قبل از سن زبان‌آموزی صورت گیرد.

Title: تشخیص، ارزیابی و درمان اختلال هماهنگی رشدی: مقاله مروری

http://jrrs.mui.ac.ir/index.php/jrrs/article/view/857

مقدمه: در این مقاله شواهد و مستندات درباره تعریف، تشخیص و درمان اختلال هماهنگی رشدی ارایه می‌شود. مواد و روش‌ها: بعد از جستجوی سیستماتیک در سایت‌های معتبر مانند PubMed، Google Scholar، Proquest، Science Direct و Cochrane Library مقالات مرتبط با اختلال هماهنگی رشدی انتخاب شدند. پس از معرفی اختلال، تاریخچه نام‌گذاری، دلایل شناسایی به موقع و در نهایت برخی از روش‌های درمانی بیان گردید. یافته‌ها: به دلیل نبود ابزار ارزیابی استاندارد و آگاهی کافی در خصوص اختلال هماهنگی رشدی، تشخیص این اختلال با مشکل روبرو است. این اختلال با گذر زمان از بین نمی‌رود و پیامدهای ثانویه منفی بر جای می‌گذارد. نتیجه‌گیری: برای تشخیص به موقع و درمان مناسب باید پژوهش‌های بیشتری صورت گیرد و کارشناسان حوزه سلامت نیز باید دانش بیشتری در این زمینه کسب نمایند.
